# Relationship between timing of achieving energy sufficiency and clinical outcomes in critically ill patients

**DOI:** 10.3389/fnut.2025.1565394

**Published:** 2025-06-30

**Authors:** Xiangfeng Yue, Xiaoxi Zhu, Yongchun Li, Xuemin Huang, Quanjun Lyu

**Affiliations:** ^1^Department of Pharmacy, The First Affiliated Hospital of Zhengzhou University, Zhengzhou, China; ^2^Department of Clinical Nutrition, Children’s Hospital Affiliated to Zhengzhou University, Zhengzhou, China; ^3^Department of Clinical Nutrition, Henan Children’s Hospital, Zhengzhou Children’s Hospital, Zhengzhou, China; ^4^Center for Disease Control and Prevention, Zhengzhou, China; ^5^Department of Nutrition and Food Hygiene, College of Public Health, Zhengzhou University, Zhengzhou, China

**Keywords:** energy sufficiency timing, critical illness, nutritional support, 60-day mortality, restricted cubic spline analysis energy sufficiency timing, restricted cubic spline analysis

## Abstract

**Background and aim:**

Malnutrition is a critical challenge in intensive care unit (ICU) patients, with the timing of energy sufficiency being a key yet debated factor in nutritional support. This study aimed to investigate the association between the timing of achieving energy sufficiency (defined as ≥70% of daily energy targets, 17.5 kcal/kg/day) in critically ill patients and their clinical outcomes, providing evidence-based guidance for ICU nutritional protocols.

**Methods:**

In this prospective observational study, adult patients admitted to the ICU for ≥3 days were stratified into three groups based on the time to achieve energy sufficiency: early (≤3 days), middle (4–7 days), and late (>7 days). Clinical outcomes, including in-hospital mortality, 60-day mortality, ICU length of stay, and gastrointestinal complications, were compared across groups. Cox proportional hazards regression models were used to assess the independent association between energy sufficiency timing and mortality, while restricted cubic spline (RCS) analysis explored nonlinear dose–response relationships using days to energy sufficiency as a continuous variable. Statistical analyses were performed using SPSS 25.0 and R 4.2.3 (two-tailed tests, *α* = 0.05).

**Results:**

A total of 826 critically ill patients were initially screened, with 584 meeting the predefined inclusion and exclusion criteria and ultimately enrolled in this study. The middle-group patients (achieving energy sufficiency at 4–7 days) demonstrated the lowest in-hospital mortality (15.6%) and 60-day mortality (28.5%), significantly lower than the late group (32.0 and 49.0%, respectively; *p* < 0.001). After adjusting for confounders (age, BMI, disease severity, etc.), both early and middle energy sufficiency remained independent protective factors against 60-day mortality (HR = 0.398 and 0.399, respectively; *p* < 0.001). RCS analysis revealed a nonlinear dose–response relationship: mortality decreased with delayed energy sufficiency up to day 6, after which mortality risk significantly increased (*p* < 0.001 for overall correlation; inflection point at day 6).

**Conclusion:**

The timing of achieving energy sufficiency (17.5 kcal/kg/day) is significantly associated with 60-day mortality in ICU patients. Combining RCS-derived inflection point (day 6) and intergroup comparisons, the optimal window for achieving energy sufficiency appears to be 4–6 days post-ICU admission, balancing metabolic stability and tissue repair needs while avoiding early overfeeding risks.

## 1 Introduction

Critical illness, triggered by major trauma, extensive burns, or life-threatening diseases, induces a state of severe physiological stress characterized by hypercatabolism and insulin resistance (1). This metabolic derangement accelerates the breakdown of skeletal muscle and adipose tissue, leading to progressive wasting, functional decline, and heightened vulnerability to multi-organ failure, nosocomial infections, and critical illness–related debility (2, 3). Concurrently, factors such as disease-induced anorexia, psychological stress (e.g., depression, anxiety), and clinical interventions (e.g., tracheal intubation, preoperative fasting, gastrointestinal dysfunction) collectively restrict oral and enteral intake, often resulting in energy deficits and increasing the risk of malnutrition (4–6). Notably, malnutrition affects 30–50% of ICU patients, prolonging hospital and ICU stays while escalating the risk of complications (e.g., infections, ICU-acquired weakness) and mortality (7–9).

Malnutrition management in critically ill patients remains a major clinical challenge, with nutritional support strategies—including timing of initiation, delivery route, and macronutrient targets—being critical determinants of outcomes (10, 11). Despite guidelines from organizations like the Society of Critical Care Medicine (SCCM) and ASPEN recommending early achievement of energy goals (within 24–48 h) for high-risk patients (12), early isocaloric feeding has been associated with refeeding syndromes, including hyperglycemia, hepatic steatosis, and increased infection risk (13). While combined enteral nutrition (EN) and parenteral nutrition (PN) improves energy delivery compared to EN alone, it does not consistently affect 30-day mortality or length of stay (14). For example, Arabi et al.’s (15) RCT showed similar 90-day mortality between hypocaloric (≤14 kcal/kg/day) and full-caloric (≥70% target) feeding groups, but fewer infections in the hypocaloric arm. Subsequent studies by Heidegger et al. (16) and Allingstrup et al. (17) have reported conflicting results when comparing indirect calorimetry-guided individualized nutrition (e.g., 25 kcal/kg/day) with standardized protocols. These inconsistencies highlight unresolved questions about the optimal timing and dosage of energy delivery. To address this gap, our study hypothesized that the timing of achieving energy sufficiency (≥70% of target) would influence 60-day mortality in ICU patients. By systematically evaluating the association between energy sufficiency timing and clinical outcomes, we aim to provide evidence for personalized nutritional protocols in critical care.

## 2 Method

### 2.1 Participants

This prospective observational study enrolled adult patients admitted to the intensive care unit (ICU) of the First Affiliated Hospital of Zhengzhou University between December 2020 and October 2021. Inclusion criteria were: (1) diagnosis of critical illness; (2) aged 18–90 years; (3) ICU stay ≥72 h. Exclusion criteria included contraindications to nutritional support, severe hepatic failure, extracorporeal membrane oxygenation (ECMO) support, severe mental disorders, secondary shock, pregnancy/lactation, participation in other trials, or refusal to consent. Patients were stratified into three groups based on the time to achieve energy sufficiency (defined as ≥70% of daily energy targets, 17.5 kcal/kg/day): early (≤3 days), middle (4–7 days), and late (>7 days).

The study was approved by the ethics committee of the First Affiliated Hospital of Zhengzhou University (2021-KY-0023-001), and written informed consent was obtained from all participants or their legal representatives. All the data used for analysis was anonymous.

### 2.2 Data collection

In this study, to minimize measurement bias, a single trained researcher systematically collected data using a standardized case report form (CRF) via the electronic medical record (EMR) system. Data were prospectively extracted from patient charts, daily nutrition prescriptions, and nursing records.

#### 2.2.1 Baseline data

The following data were recorded in the baseline: patient general information (sex, age, height and weight), nutritional status, and severity of disease. Nutritional status was evaluated using the Nutritional Risk Screening 2002 score (NRS 2002) and modified Nutrition Risk in the Critically Ill score (mNUTRIC) (7, 9); disease severity was assessed via the Acute Physiology and Chronic Health Evaluation II (APACHE II) score and Sequential Organ Failure Assessment (SOFA) score (1, 3).

#### 2.2.2 Nutritional exposure data

Nutritional parameters were recorded daily until hospital discharge or up to day 14 in the ICU, including:

Support modalities: oral feeding, enteral nutrition (EN), parenteral nutrition (PN), or combined EN+PN.
Energy sources: glucose dose (via PN/EN), type and dose of EN preparations, type and dose of PN preparations, and oral intake quantified by direct weighing of uneaten food (precision ± 5 g).
Energy intake calculation: Daily energy intake was computed as the sum of calories from oral feeding, EN, and PN, based on nutrient composition tables. According to the ESPEN guidelines, hypocaloric or underfeeding is an energy administration below 70% of the defined target (20–25 kcal/kg/day). So we consider a target of energy intake ≥70% of the ESPEN-recommended target as energy sufficiency (18). Patients were stratified into:
Early group: energy sufficiency achieved within ≤3 days (first 3-day average ≥17.5 kcal/kg/day).
Middle group: energy sufficiency achieved on days 4–7 (first 7-day average ≥17.5 kcal/kg/day, with days 1–3 < 17.5 kcal/kg/day).
Late group: energy sufficiency achieved after >7 days (first 14-day average ≥17.5 kcal/kg/day, with days 1–7 < 17.5 kcal/kg/day).

#### 2.2.3 Outcome data

Primary and secondary outcomes included:

Mortality: in-hospital mortality and 60-day all-cause mortality.
Length of stay: ICU duration and total hospital stay (days).
Gastrointestinal (GI) complications: diarrhea: defined as ≥3 loose stools/day for ≥2 consecutive days; GI intolerance: documented vomiting, abdominal distension, or gastric residual volume >200 mL (5, 13).

### 2.3 Statistical methods

We first described basic information, nutritional status, disease severity, and patient outcomes in the study population, and then we analyzed differences in clinical outcomes between patients with different timing of achieving energy sufficiency. For quantitative data, comparisons between groups of multiple independent samples were analyzed using one-way analysis of variance (ANOVA) if normality was satisfied and variances were chi-squared, otherwise the Kruskal-Wallis test was used. For qualitative data, the *X*^2^ test was used for intergroup comparisons, and Fisher’s exact probability method was used if the test conditions were not met.

We used a Cox proportional hazard regression model to explore the linear relationship between different timing of achieving energy sufficiency and patient survival outcomes. However, in actual clinical practice, the efficacy of nutritional interventions may exhibit a threshold effect or inflection point phenomenon. Therefore, we employed restricted cubic spline (RCS) analysis to explore the nonlinear association between the specific number of days to achieve energy sufficiency in the ICU and 60-day mortality among patients who reached energy sufficiency. Subgroup analyses were subsequently performed to further explore whether outcomes differed significantly by age and gender. RCS were plotted using R 4.3.2, and all other analyses were performed using SPSS 25.0 with two-sided tests at a test level of *α* = 0.05.

## 3 Results

### 3.1 Basic information about the study subjects

**Figure 1:**
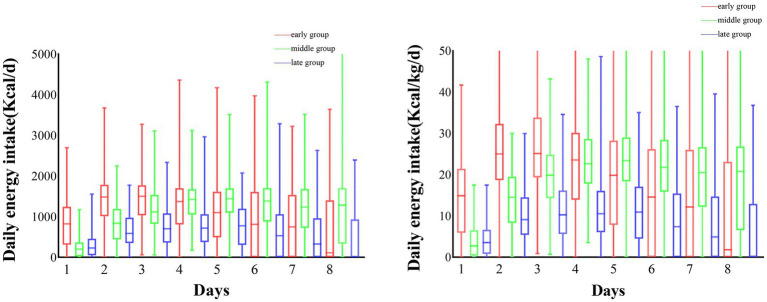
The average daily energy intake of the patient.

Overall, a total of 584 patients were included in this study, 204 patients in the early group, 186 in the middle group, and 194 in the late group. we compared baseline characteristics between groups at different times of achieving energy adequacy, and the results showed that there were no statistically significant differences between the three groups in terms of gender, age, mNUTRIC score, NRS 2002 score, and APACHE II score (*p* > 0.05), and statistically significant differences in terms of BMI, SOFA score, mode of nutritional support, and daily energy intake (*p* < 0.05). For specific details, please refer to Table 1. The average daily energy intake of the three groups of patients is shown in Figure 1.

**Table 1:** Basic information about patients.

Basic information	Total (*n* = 584)	Timing of achieving energy sufficiencs			χ^2^/*Z*	*p* value
		Early group (*n* = 204)	Middle group (*n* = 186)	Late group (*n* = 194)		
Sex, *n* (%)						
Man	355 (60.8)	114 (32.1)	114 (32.1)	127 (35.8)	3.859	0.145
Woman	229 (39.2)	90 (39.3)	72 (31.4)	67 (29.6)		
Age (year), M (P25, P75)	62 (51, 71)	63.00 (55.00, 72.00)	60.50 (49.00, 70.00)	63.00 (50.00, 71.00)	2.404	0.301
BMI (kg/m^2^), M(P25, P75)	22.04 (19.38, 24.97)	20.76 (18.22, 23.88)	21.48 (18.81, 24.42)	23.69 (21.48, 27.17)	62.347	<0.001
Admission diagnosis category, *n* (%)					16.376	<0.001
Medicine	502 (86.0)	187 (37.3)	164 (32.7)	151 (30.1)		
Postoperative & Trauma	82(14.0)	17(20.7)	22(26.8)	43(52.4)		
Complications, *n* (%)					127.257	<0.001
Hypertension	190 (32.5)	67 (35.5)	52 (27.4)	71 (37.4)		
Diabetes	110 (18.8)	32 (29.1)	34 (30.9)	44 (40.0)		
Chronic obstructive pulmonary disease	49 (8.4)	21 (42.9)	15 (30.6)	13 (26.5)		
Respiratory failure	203 (34.8)	69 (34.0)	75 (36.9)	59 (29.1)		
Coronary heart disease	60 (10.3)	16 (26.7)	13 (21.7)	31 (51.7)		
Cardiac failure	36 (6.2)	11 (30.6)	10 (27.8)	15 (41.7)		
Cerebrovascular disease	34 (5.8)	12 (35.3)	11 (32.4)	11 (32.4)		
Tumour	80 (13.7)	28 (35.0)	28 (35.0)	24 (30.0)		
Others	27 (4.6)	11 (40.7)	6 (22.2)	10 (37.0)		
Nutritional status, *n* (%)						
mNUTRIC score					4.686	0.096
≥5 score	277 (38.9)	132 (47.7)	117 (42.2)	108 (39.0)		
<5 score	357 (61.1)	69 (19.3)	71 (19.9)	87 (24.4)		
NRS 2002 score					9.226	0.056
≥5 score	251 (43.0)	98 (39.0)	82 (54.3)	71 (47.0)		
3–4 score	267 (45.7)	80 (30.0)	82 (30.7)	105 (39.3)		
<3 score	66 (11.3)	26 (39.4)	22 (33.3)	18 (27.3)		
Severity of illness, M (P25, P75)						
APACHEII score	15.00 (11.00, 21.00)	14.00 (10.00, 20.00)	15.00 (11.00, 21.00)	16.50 (11.00, 23.00)	5.261	0.072
SOFA score	5.00 (3.00, 7.00)	4.00 (2.00, 6.00)	5.00 (3.00, 7.00)	6.00 (4.00, 8.00)	35.332	<0.001
Nutritional support mode, *n* (%)					49.318	<0.001
EN	122 (20.9)	43 (35.2)	46 (37.7)	33 (27.0)		
PN	121 (20.7)	33 (27.3)	23 (19.0)	65 (53.7)		
EN+PN	210 (36.0)	63 (30.0)	86 (41.0)	61 (29.0)		
Oral feeding	131 (22.4)	64 (48.9)	35 (26.7)	32 (24.4)		
Energy intake						
Daily energy intake (kcal/d), M (P25, P75)	1056.58 (806.37, 1301.62)	1233.91 (978.22, 1470.23)	1187.67 (981.98, 1329.56)	735.33 (528.88, 905.47)	212.875	<0.001
Daily energy intake (kcal/kg/d), M (P25, P75)	17.63 (12.78, 21.24)	20.70 (17.81, 25.79)	19.12 (17.54, 21.48)	11.23 (8.09, 13.56)	337.227	<0.001
Daily energy intake (% of requirement), M (P25, P75)	100.74 (73.04, 121.39)	118.30 (101.81, 147.39)	108.28 (100.20, 122.73)	64.14 (46.26, 77.49)	337.227	<0.001

NRS 2002 score: <3 defined as no nutritional risk, ≥3 and <5 defined as risk of nutritional, ≥5 defined as high nutritional risk; mNUTRIC score: <5 defined as low nutritional risk, ≥5 defined as high nutritional risk.

### 3.2 Relationship between timing of achieving energy sufficiency and clinical outcomes

We compared clinical outcomes between groups at different times of achieving energy sufficiency, as shown in Table 2. There were no statistically significant differences in the incidence of gastrointestinal intolerance and diarrhea among the three groups (*p* > 0.05), and there were statistically significant differences between in-hospital mortality and 60d mortality (*p* < 0.001). Patients in the late group exhibited the highest in-hospital mortality and 60-day mortality, followed by those in the early group, while patients in the middle group had the lowest mortality rates. In terms of ICU length of stay and hospital duration, mid-term group patients had the longest ICU and hospital stays, followed by early group patients, with late group patients demonstrating the shortest durations.

**Table 2:** Clinical outcomes for achieving energy sufficiency at different times.

Clinical outcomes	Timing of achieving energy sufficiencs			χ^2^/*Z*	*p* value
	Early group	Middle group	Late group		
Gastrointestinal intolerance, *n* (%)	19 (9.8%)	24 (13.3%)	30 (17.5%)	4.628	0.099
Diarrhea, *n* (%)	4 (2.1%)	8 (4.4%)	11 (6.5%)	4.339	0.114
In-hospital mortality, *n* (%)	36 (17.6%)	29 (15.6%)	62 (32.0%)	18.045	<0.001
60-d mortality, *n* (%)	63 (30.9%)	53(28.5%)	95 (49.0%)	28.885	<0.001
Length of stay in ICU, M (P25, P75)	7.00 (4.50, 13.00)	11.00 (8.00, 16.00)	6.50 (4.00, 10.00)	50.476	<0.001
Length of stay in hospital, M (P25, P75)	14.00 (7.00, 24.50)	15.00 (10.00, 24.00)	10.00 (6.00, 15.00)	33.584	<0.001

After that, we included the timing of achieving energy sufficiency as a variable in the COX regression model and the results are shown in Table 3 (Including sensitivity analysis). Using the late group as a reference, achieving energy sufficiency in the early (HR = 0.469, 95% CI: 0.340, 0.646, *p* < 0.001) and middle (HR = 0.441, 95% CI: 0.315, 0.618, *p* < 0.001) periods were both protective factors for 60-day mortality. After adjusting for confounders such as sex, age, BMI, nutritional status, and disease severity, early group and middle group remained protective factors for 60d death.

**Table 3:** Cox regression with 60-day mortality.

*HR*/Different timing		Early group	Middle group	Late group
Total patients	*HR_1_*	0.469 (0.340, 0.646)	0.441 (0.315, 0.618)	1
	*P*	<0.001	<0.001	
	*HR_2_*	0.398 (0.280, 0.566)	0.399 (0.281, 0.567)	1
	*P*	<0.001	<0.001	
Sensitivity analysis (medicine patients)	*HR_1_*	0.442 (0.316, 0.620)	0.429 (0.300, 0.612)	1
	*P*	<0.001	<0.001	
	*HR_2_*	0.407 (0.282, 0.587)	0.393 (0.272, 0.569)	1
	*P*	<0.001	<0.001	

*HR_1_* is an unadjusted variable, *HR_2_* is a variable adjusted for age, BMI, Admission diagnosis category, NRS scores, mNUTRIC scores, APACHE II scores, and SOFA scores.

### 3.3 Dose–response relationship between specific timing of achieving energy sufficiency and 60-day mortality

**Figure 2:**
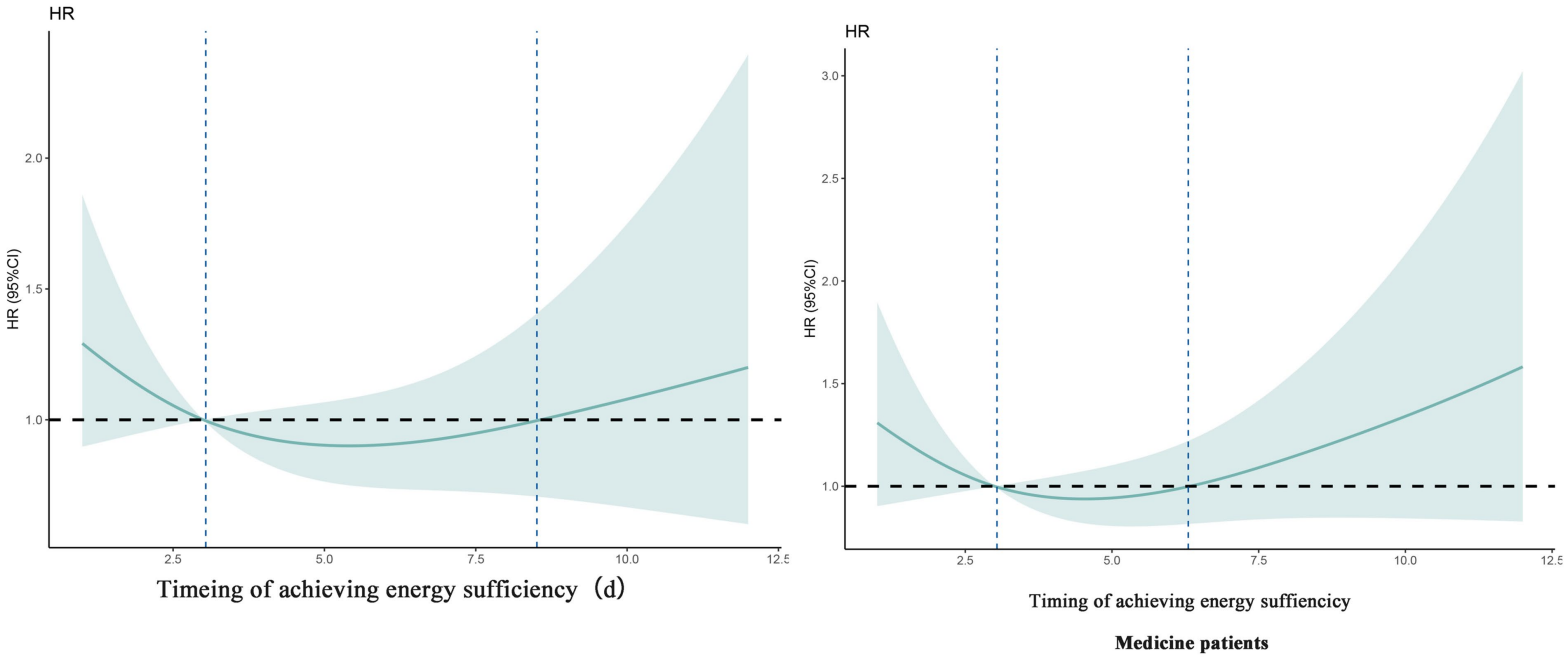
Dose–response relationship between specific time of achieving energy sufficiency and 60-day mortality (overall patient and sensitivity analysis).

There was an overall correlation between the specific timing of patient achieving energy sufficiency in the ICU and 60-day death, but there was no nonlinear association (*P_total_* < 0.001, *P_nonlinear_* = 0.186), as shown in Table 4. The specific trend of the association is shown in Figure 2. The graph suggests that the later the patient achieves energy sufficiency from ICU admission to day 6, the lower the 60d risk of death, and the later the time after day 6, the higher the 60d risk of death. The sensitivity analysis for medicine patients also showed the same result.

**Table 4:** Dose–response relationship between timing of achieving energy sufficiency 60-day death.

Variable	*n*	Global correlation			Nonlinear correlation		
		*χ* ^2^	Df	*p*	*χ* ^2^	Df	*p*
Overall patient	389	29.905	9	<0.001	1.250	1	0.186
Sensitivity analysis							
Medicine patients	350	24.198	9	<0.001	2.773	1	0.095
Subgroup analysis							
Age (year)							
18–59	179	22.588	9	0.007	4.362	1	0.037
≥60	210	12.084	9	0.209	0.001	1	0.970

**Figure 3:**
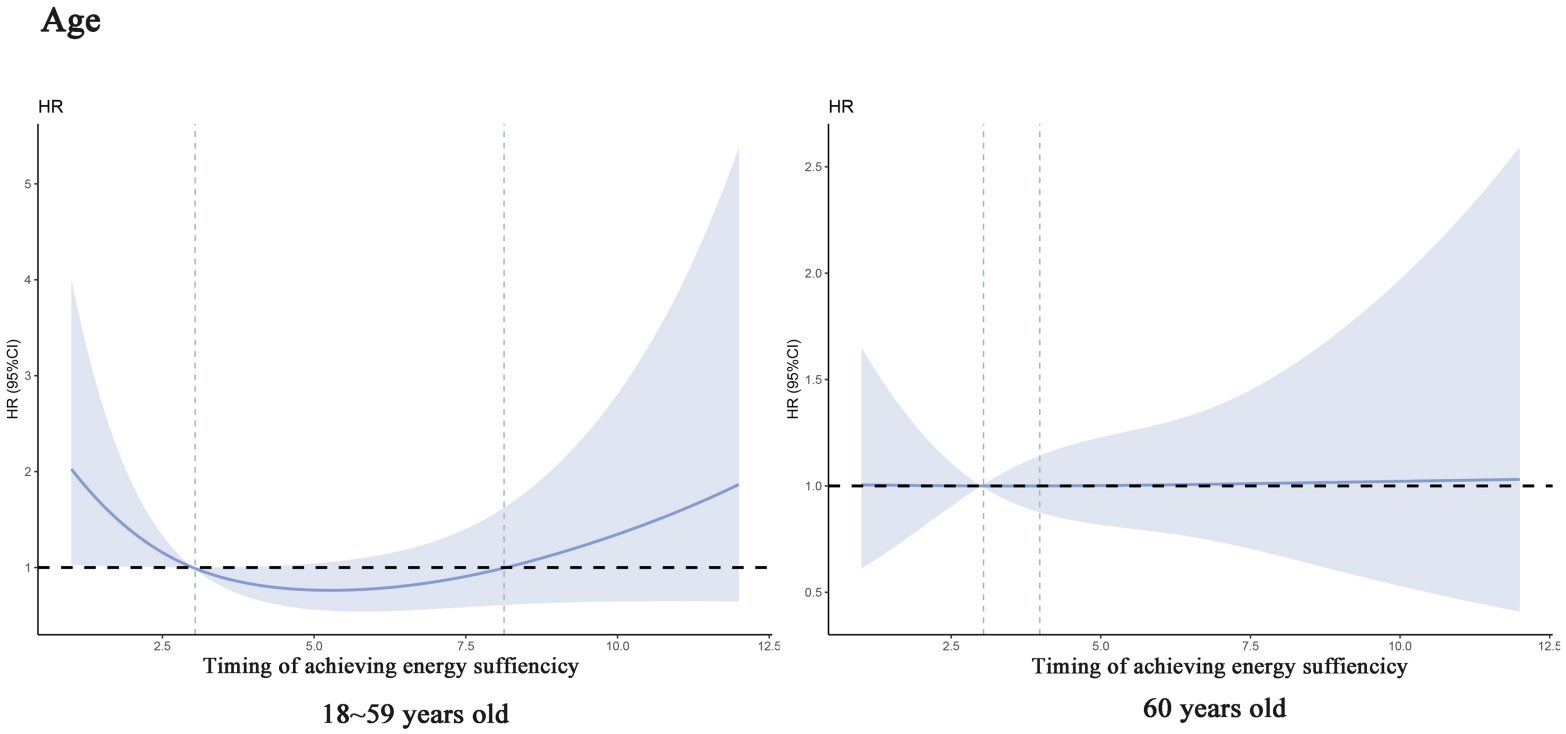
Dose–response relationship between specific time of achieving energy sufficiency and 60-day mortality (subgroup analysis).

In addition, we performed subgroup analyses which showed a linear association between timing of achieving energy sufficiency and 60-day death in men patients (*P_total_* = 0.009, *P_nonlinear_* = 0.930), whereas there was a significant non-linear association between timing of achieving energy sufficiency and 60-day death in patients aged 18–59 years (*P_total_* = 0.007, *P_nonlinear_* = 0.037), and the trend of the association is shown in Figure 3.

## 4 Discussions

In this study, 584 critically ill patients were categorized into early, middle, and late groups based on the timing of achieving energy sufficiency (≥70% of the ESPEN-recommended target, 17.5 kcal/kg/day). Intergroup comparison revealed that middle-group patients exhibited the lowest in-hospital mortality (15.6%) and 60-day mortality (28.5%), significantly lower than the late group (32.0 and 49.0%, respectively; *p* < 0.001). While the middle group had longer ICU and hospital stays, this may reflect a balance between metabolic stabilization and active tissue repair. Mechanistically, initiating nutritional support at 4–7 days aligns with the decline of the acute inflammatory phase (e.g., reduced IL-6 and TNF-*α* levels), which restores insulin sensitivity and enhances energy utilization for healing rather than catabolic processes (2, 19). This timing also avoids the risks of early overfeeding, such as hyperglycemia and hepatic steatosis, while preventing irreversible muscle wasting (typically occurring after 7–10 days of inadequate nutrition) (13, 19).

Multivariate Cox regression confirmed that both early (≤3 days) and middle (4–7 days) energy sufficiency were independent protective factors against 60-day mortality (adjusted HR = 0.398 and 0.399, respectively; *p* < 0.001). Restricted cubic spline (RCS) analysis further revealed a nonlinear dose–response relationship, with 60-day mortality decreasing up to day 6 and significantly increasing thereafter (p < 0.001 for overall correlation), supporting a critical threshold at day 6. This finding aligns with Heidegger et al.’s (16) RCT, where initiating full-energy parenteral nutrition on day 4 reduced hospital-acquired infections, and Matejovic et al.’s (20) study showing that energy deficits after day 5 were strongly associated with mortality.

Notably, our results contrast with a Chinese study reporting no impact of early energy sufficiency on outcomes (21). This discrepancy may arise from study design differences, such as inclusion of predominantly surgical patients (with lower metabolic stress than medical ICU patients), use of higher energy targets (25 kcal/kg/day vs. our 17.5 kcal/kg/day), or reliance on enteral nutrition alone (17, 21, 22). Indeed, the middle group in our study had a higher proportion of combined enteral + parenteral nutrition (46.2%), which may optimize energy delivery while preserving gut barrier function (10, 14).

Subsequently, we conducted a subgroup analysis stratified by age, and the results showed that patients <60 years showed a stronger association between delayed energy sufficiency and mortality, while the trend was relatively flat in patients over 60 years of age. This phenomenon may be related to the differences in physiological reserves, metabolic characteristics, and disease stress response in different age groups. <60-year-old patients are more likely to be in the prime of life, and the stress response triggered by critical illnesses is more intense, manifesting itself in higher stress-induced resting energy expenditure (REE, up to 50–100% above baseline) and faster rate of muscle catabolism (3). At this time, insufficient energy intake can rapidly exacerbate malnutrition, leading to decreased immune function and organ dysfunction, which can lead to a bad outcomes (19). In contrast, ≥60-year-old patients often have sarcopenia, a low basal metabolic rate, and may be in a chronic inflammatory state, exhibit “pseudo-normalized” energy requirements due to sarcopenia and chronic inflammation, blunting short-term mortality impacts (23).

Despite numerous observational studies and randomized controlled trials (RCTs) conducted over the past decade, the optimal timing and dosage of energy delivery in critically ill patients remain unclear. Based on our findings, we recommend providing patients with at least 17.5 kcal/kg/day of energy within 4–6 days after ICU admission. This study uniquely integrates energy intake timing with ICU stay duration to analyze their combined impact on 60-day mortality, offering novel evidence and directions for future research. Notably, while we observed a significant correlation between the timing of achieving energy sufficiency and 60-day mortality, this association should be interpreted as a potential marker of overall care quality or recovery trends rather than a definitive causal relationship. In clinical practice, nutritional strategies must be comprehensively evaluated alongside other medical interventions to account for individual variability in metabolic responses and disease severity.

Our study has several notable limitations that should be considered when interpreting the results. First, as an observational study, it inherently carries susceptibility to residual confounding, despite our application of multivariable regression models to adjust for known covariates. For instance, unmeasured factors—such as pre-ICU nutritional status, genetic predispositions to metabolic responses, and the impact of specific ICU interventions (e.g., sedation protocols, mechanical ventilation parameters)—may potentially influence both the timing of achieving energy sufficiency and clinical outcomes. Second, defining energy sufficiency as achieving 70% of the ESPEN-recommended target (17.5 kcal/kg/day) represents a simplified approach that may not capture the individualized nutritional requirements of critically ill patients. Our study did not account for dynamic fluctuations in energy expenditure caused by factors such as fever, sepsis, or surgical procedures. Third, the single-center design of the study may restrict the generalizability of our findings, as patient demographics, treatment protocols, and nutritional practices can differ across diverse healthcare settings.

To address these limitations, several promising future research directions deserve dedicated exploration. First, randomized controlled trials (RCTs) are pivotal to establish a causal link between the timing of achieving energy sufficiency and clinical outcomes. Such trials should systematically compare contrasting nutritional strategies such as early initiation versus delayed escalation of full-energy support—while employing sophisticated methodologies to control for confounding variables, thereby strengthening the validity of causal conclusions. Second, the advancement of personalized nutrition protocols represents a critical priority. Future studies could integrate real-time assessments of energy expenditure (e.g., via indirect calorimetry) and dynamic biomarker monitoring (e.g., serum prealbumin, C-reactive protein) to tailor nutritional interventions to the unique metabolic profiles of individual patients. Especially considering the heterogeneity in metabolic responses among critically ill populations, as it would enable adaptive nutritional support that aligns with fluctuating physiological demands (e.g., fever, sepsis, or surgical trauma). Additionally, multicenter studies with large, diverse cohorts are indispensable to enhance the generalizability of these findings. By enrolling patients across geographically and demographically varied healthcare settings, such research could validate the optimal window for achieving energy sufficiency (e.g., 4–6 days post-ICU admission) in populations with differing baseline characteristics, comorbidities, and institutional protocols. This would mitigate the single-center limitation of the present study and foster the development of universally applicable nutritional guidelines.

## 5 Conclusion

Our findings suggest that the timing of energy sufficiency (70% of energy goal, i.e., 17.5 kcal/kg/d) in critically ill patients is closely related to 60-day mortality, and the optimal window for achieving energy sufficiency is 4–6 days. This finding provides key evidence for the precise formulation of nutritional support protocols in the ICU.

## Data Availability

The raw data supporting the conclusions of this article will be made available by the authors, without undue reservation.
